# The influence of neighborhood quality on tourism in China: Using Baidu Street View pictures and deep learning techniques

**DOI:** 10.1371/journal.pone.0276628

**Published:** 2022-11-03

**Authors:** Jieping Chen, Zhaowei Wu, Shanlang Lin

**Affiliations:** School of Economics and Management, Tongji University, Shanghai, China; Monash University, AUSTRALIA

## Abstract

Previous studies have investigated the determinants of urban tourism development from the various attributes of neighborhood quality. However, traditional methods to assess neighborhood quality are often subjective, costly, and only on a small scale. To fill this research gap, this study applies the recent development in big data of street view images, deep learning algorithms, and image processing technology to assess quantitatively four attributes of neighborhood quality, namely street facilities, architectural landscape, green or ecological environment, and scene visibility. The paper collects more than 7.8 million Baidu SVPs of 232 prefecture-level cities in China and applies deep learning techniques to recognize these images. This paper then tries to examine the influence of neighborhood quality on regional tourism development. Empirical results show that both levels of street facilities and greenery environment promote tourism. However, the construction intensity of the landscape has an inhibitory influence on the development of tourism. The threshold test shows that the intensity of the influence varies with the city’s overall economic level. These conclusions are of great significance for the development of China’s urban construction and tourism economy, and also provide a useful reference for policymakers. The methodological procedure is reduplicative and can be applied to other challenging cases.

## Introduction

The improvement of neighborhood quality is closely related to the development of urban tourism [1]. Good neighborhood quality includes not only high-level public service, but also the city’s unique architectural style, beautiful ecological environment, convenient street facilities, etc. All of these can bring the visitors a comfortable sightseeing experience, improve the attractiveness for tourists, and significantly strengthen the city’s competitiveness in tourism [2] and other industries. However, scholars have argued that tourism as an integral part of city marketing and development hasn’t gained sufficient attention in urban research [3]. Therefore, this paper attempts to objectively evaluate the neighborhood quality and explore its influence on urban tourism development.

Previous studies have investigated the determinants of urban tourism development from the perspective of neighborhood context or environment. However, most of them are qualitative analyses focusing on the concept’s interpretation or belong to general experience analysis or case study. Whether these research results can provide generalized implications for other cities is unknown. Due to the insufficient quantitative analysis, studies have not yet led to a consensus about the specific relationship between neighborhood quality and the tourism industry. Traditional methods to assess neighborhood quality are often subjective, costly, and only on a small scale [4]. As Long [5] asserts, it is difficult for researchers to have large-scale micro-data at the street level to measure neighborhood quality. In addition, previous evaluations of neighborhood quality, such as questionnaires, on-site surveys, and field observation, are time-consuming and easily cause bias [6]. Therefore, the existing literature is limited on the objective and effective evaluation of the neighborhood quality, which has become the prerequisite for further exploring its impact on regional tourism development.

To overcome the limitations of the previous evaluation method, this study applies the recent development in big data of street view images, machine learning, and image processing technology to effectively measure neighborhood quality [7]. The purpose of this paper is to examine the influence of neighborhood quality on regional tourism development and to provide a feasible method of combining SVPs and deep learning algorithms for quantifying the neighborhood quality for large number of cities. The paper firstly collects more than 7.8 million Baidu SVPs obtained from 232 prefecture-level cities in China. There are altogether 285 prefecture-level administrative cities in China. Due to the data availability of SVPs in some distant cities, this paper discusses 232 cities of them as research sample. Then, we apply deep learning techniques to recognize these images and quantitatively measure four attributes of the neighborhood quality, namely, street facilities, architectural landscape, green or ecological environment, and scene visibility. The paper then conducts an empirical regression analysis between neighborhood quality and tourism development. Moreover, this paper further explores the threshold effect considering the heterogeneity in different cities. The empirical results show strong support for the development of China’s urban construction and tourism economy, and also provide a useful reference for policy makers.

The main contributions of this paper are: (1) It introduces the concept of neighborhood quality in the research field of economics and explores its relationship with urban tourism. This study broadens the theoretical and empirical research perspective about the tourism industry in economics and urban study. (2) Applying the combined method of deep learning and large-scale street view at a pedestrian level, we quantitatively analyze and measure four attributes of neighborhood quality. It advances previous qualitative research in urban planning. In this regard, our study enriches the research method in related fields and introduces the possibility of applying SVPs and deep learning methods for urban assessment.

The rest of this paper is arranged as follows: Section 2 introduces the related literature review. Section 3 describes the materials and methods for our analysis. Section 4 reports empirical results and further analysis. Conclusion and discussion are presented in the last section.

## Literature review

This study is based on two main branches of literature. One is about the relationship between neighborhood quality and urban tourism. And the other is the methodological application of SVPs and deep learning techniques for measuring street landscapes and neighborhood quality.

### Relationship between neighborhood quality & tourism

In the early stage of industrial society, tourism and cities deviated from each other. The urban diseases brought by high-speed industrialization force people to escape from the city and return to nature. Cities have become an important source of tourists. After that, with the improvement of the urban environment and the continuous improvement of equipment and facilities in the city, the city has the functions of management, reception, leisure, and entertainment. Tourism begins urbanization [8], and the urban tourism industry enters a competitive growth stage [9]. Resulting of the rapid development of transportation and Internet technology, the competition among cities has focused on the local quality while the neighborhood is the starting point for improving local quality. Previous studies have shown that good neighborhood quality can significantly enhance a city’s tourism competitiveness [2]. From the perspective of environmental psychology and behavior science, neighborhood quality usually includes four attributes: street facilities, the city’s architectural landscape, greenery or ecological environment, and scene visibility.

In the research on neighborhood quality and tourism development, scholars mostly focus on the influence of street style attributes of the neighborhood quality on tourism. Street layout with high density and strong connectivity broadens the available options of urban transportation and increases the competitiveness of urban tourism [10]. Fluent road traffic and perfect road facilities can provide tourists with a convenient transportation experience [11]. Meanwhile, road traffic with high mobility has greatly improved accessibility [12]. However, it is accompanied by high accidents and severe pollution. Differently, narrow pedestrian roads provide visitors with more opportunities for walking, resting, and sightseeing [13].

Referring to the architectural style, the appropriate building layout creates friendly and integrated visiting surroundings, thus bringing tourists a comfortable sightseeing experience. In contrast, a dense building layout produces pressure [11]. For example, Tyrväinen and others [14] study the impact of building density on the subjective psychological feelings of tourists and find that the highly-dense building layout in resorts significantly reduces the visual scope of green space and natural landscape and thus causes psychological pressure on tourists. In a similar study, Ren [15] argues in a study of Xi ’an city that the high-density multi-storey buildings can block the sights of the tourists and negatively affect their mood.

Therefore, excellent urban landscape designers usually employ various plants in the busy downtown area to enhance the greenery degree of the city and to relax the tourists from the dense and high buildings [16]. Urban designers tend to apply greenery, which can reduce the sense of pressure from high building density [17]. The visibility of vegetation can improve the safety perception of urban areas and bring tourists an easy and pleasant sightseeing experience [16]. In the study about Chinese cities, Fok [18] discovers that improving the urban greenery coverage rate in Hong Kong can promote the construction of its own urban brand and reputation, thus enhancing the competitiveness of urban tourism. The studies about foreign cities have concluded that urban greenery creates more robust ecosystems, and forest landscape plays a vital role in tourism. Therefore, appropriate management of urban greenery helps enhance urban tourism [19–21].

In addition, the street enclosure formed by the building as the main body also affects tourists’ sense of security, comfort, and satisfaction. It then influences tourism development in the city [22, 23]. Street’s spatial enclosure decides the scale size perceived by visitors [24], and the sky ratio affects their sight [25]. Both relate directly to the scene visibility and ultimately influence visitors’ emotional pleasure and pressure, thus their willingness to stay.

Altogether, the existing studies mostly emphasize the influence of a particular attribute of the neighborhood quality on the tourism industry while neglecting comprehensive analysis taking all main elements into one framework simultaneously. Moreover, the available research is limited to the study of one specific city or case study which lacks the reliability of quantitative analysis results. As a result, the conclusions may not have reference value for other cities considering cities’ heterogeneity.

### The application of SVPs & deep learning

Recently, there has been a surge in the measurement method of street landscapes in both domestic and global academia. The formation of the new data environment worldwide, the technical breakthrough of map and image processing, and the introduction of measurement technology in related fields further allow the refinement of quantitative research in street form. Among them, the emerging SVPs can provide massive data based on a pedestrian perspective as a benchmark, with the advantages of rich information, high collection efficiency, and low collection cost [26]. SVPs can thus accurately reflect various street landscapes such as sky views, building, and architectural style, greenery environment, visibility, and transportation convenience as basic elements of neighborhood quality [24, 25, 27]. Currently, Scholars have applied this technology in the evaluation of urban streets. For example, Li and others [28] construct a framework for evaluating the greenery recovery degree of urban Streets based on Google SVPs, and applies it in the evaluation of the East Village in the Manhattan District of New York. After that, Hao and Long [29] revise the study from Li and others [28] and apply the street greenery index to calculate more than 30,000 streets in Chengdu City in China. Similarly, Liu and others [30] use Baidu SVPs to evaluate the buildings on both sides of Beijing’s Fifth Ring Road streets. Steinmetz-Wood and others [31] utilize Google Street View to describe the microscale environments in a total of 129 streets from both the Montreal and Toronto regions.

In recent years, scholars have begun to broaden the use of street scenes through color band methods or machine learning techniques [32]. For example, Nagata and others [33] assess the street view walkability of communities around Tokyo city through semantic segmentation and statistical modeling for Google Street View images. Based on the generative adversarial networks (GAN) models through machine learning, Rachele and others [34] observe the influence on healthy activity from architectural features of more than 200 community buildings in Brisbane, Australia.

Although various methods exist to evaluate the micro-morphology of the city through SVPs and machine learning, existing applications revolve only around a single city or evaluate a single element [31–33, 35]. To fill the research gap, this paper utilizes micro-data at the street level in each city to scientifically evaluate the neighborhood quality from its four attributes and then carries out an empirical test.

## Materials & methods

**Fig 1:**
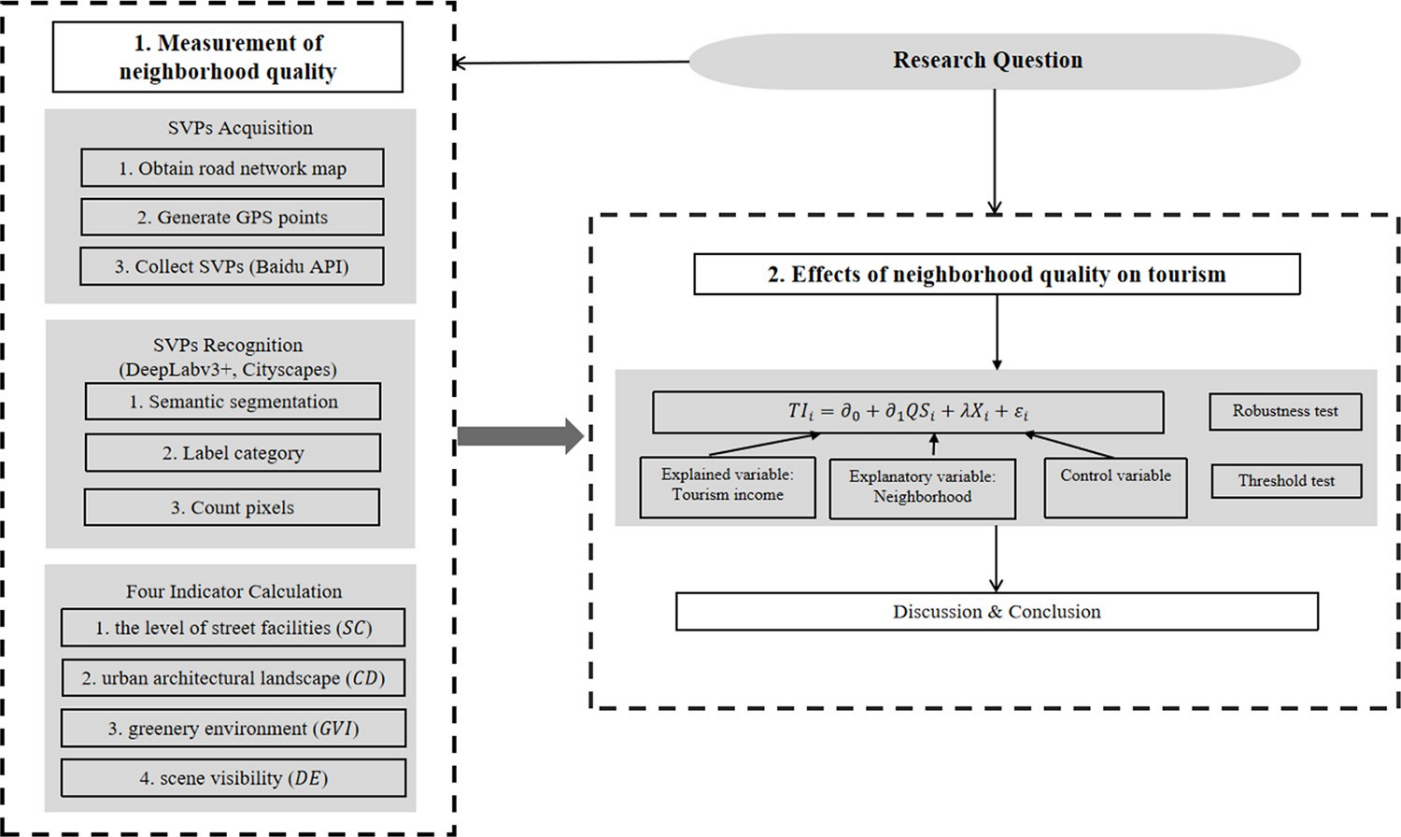
The methodological flow chart of this paper.

To examine the influence of neighborhood quality on the tourism industry, we need to quantitatively measure neighborhood quality based on the processed SVPs. Then, combined with data from China Urban Statistical Yearbook and China Environmental Statistical Yearbook, we conduct the empirical analysis with 232 major prefecture-level cities in China as the research samples. Fig 1 shows the research flow chart of this paper.

### Acquisition of SVPs

The SVPs in this study came from the Baidu Map open platform (http://lbsyun.baidu.com/). Baidu Street View and Tencent Street View have covered most cities in China and are now the primary street view data sources in mainland China. Baidu Map open platform provides free online access and an API (Application Programming Interface) for downloading Baidu SVPS (Baidu SVPs API: http://api.map.baidu.com/panorama/v2?ak = *&width = 512 &height = 256 &location = 116.313393,40.04778&fov = 180).

In recent years, data from SVPs has been widely used in many studies, providing new ideas for large-scale research at the micro-level [36–38]. Compared with the traditional urban data, the urban SVPS has the following advantages: (1) They can reflect the objective reality. Unlike questionnaires or interviews, SVPs systematically map the city’s street-level scene in detail. SVPs contain rich urban infrastructure information and provide artificial and natural landscape at the street level, which can intuitively and accurately reflect the facade information of the city. (2) Street view images come from pictures taken by street view cars, with comprehensive coverage and different perspectives. Baidu SVPs cover hundreds of cities in China, which provides a reliable data source for the research in this paper. (3) The data collection work has high efficiency and comparatively low cost. The processing logic is simple and easy for supervision and quality control. In addition, it is not limited by the weather, time, and place.

By combining GIS and the Baidu API, we apply the following steps to download the SVPs and create a dataset:

Firstly, to obtain a street network map of China from the Open Street Map (OSM) website (OSM: https://www.openstreetmap.org; China road network: https://download.geofabrik.de/asia.html). OSM is a collaborative project which produces and offers free geographic data. We obtain data about five types of roads, including the main roads (Primary), secondary roads (Secondary), tertiary roads (Tertiary), all residential roads (Residential), and service roads (Service). We then use ArcGIS software to optimize the road network data, removing streets with possible visual field duplication. Finally, we get a road network map in China.

Secondly, to generate GPS (the Global Positioning System) points. Previous studies have applied various interval criteria to obtain SVPs, such as 20 meters [39], 30 meters [40], 50 meters [32], and 100 meters [4]. Considering the time required to obtain SVP and the efficacy and validity of image content for our research purpose, we set the density of the sampling point to be 100 meters for our analysis. We set the shortest allowable distance between any two random points to ensure at least one Baidu Street View panorama every 100 meters on one road. The road network obtained in the previous step is thus sampled by the CreatePointsLines Plugin of the ArcGIS software. The GIS enables direct links through spatial connections between latitude and longitude coordinate pairs and the related datasets. As a result, we get GPS points with latitude and longitude coordinates every 100 meters along the street. The coordinates of all sample points are saved.

Thirdly, to collect SVPs. The Baidu API allows users to download SVPs by specifying required acquisition parameters, such as Size (the output size of the image in pixels), Location (coordinates of location), Fov (horizontal field of view of the image, accepted values are 0–360), Heading (compass heading of the camera, accepted values are 0–360), Pitch (up or down angle of the camera, accepted values are 0–90), etc. In order to make the obtained street view closer to the eye-level view of the pedestrians, we set "Fov" to 90° and "Heading" to 0, 90, 180, and 270, respectively. By entering the coordinates of the sampling points into a Python script, we access the Baidu Street View API and thus collect four images in each location, which can cover 360-degree horizontal surroundings. The 360-degree images can contain entire streetscapes from a specific position and are more advanced than static images in some studies. The requested SVPs were mainly captured in 2016. A tiny portion was collected in 2015 or 2017 and is basically of small roads with low pedestrian traffic and elevated highways without change. In addition, some images need to be removed in the following cases: (1) for some specified street sites, the Baidu Street View map does not record images; (2) in some visual frames, the central view is covered by objects such as trucks and buses, instead of street view. After deleting the invalid pictures, we collect ultimately more than 7.8 million Baidu SVPs (860*573 pixels) from 5,868 streets in China’s 232 prefecture-level cities from the Baidu API.

### Recognition of SVPs

To measure the neighborhood quality based on SVPs, we need to assess the various physical features of objects in each image. Specifically, there are two procedures. (1) we need to have a precise semantic segmentation for the different physical features in each image and assign a category label to each pixel in the image. (2) We use the Python tool to optimize the segmentation and count the total number of pixels in each category.

**Fig 2:**
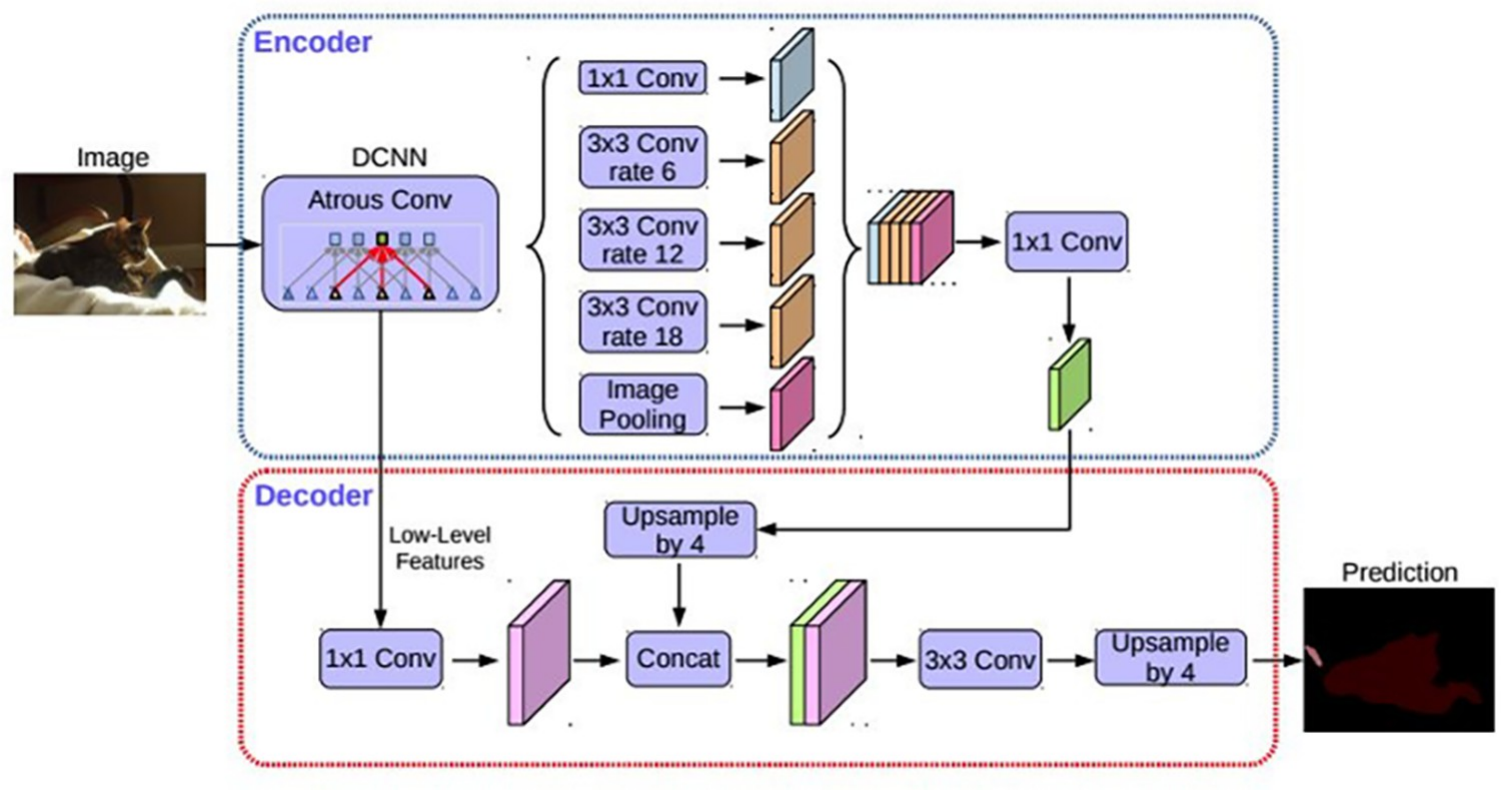
The overall architecture of the DeepLabv3+ model [44].

Image semantic segmentation uses machine learning to segment the contents of an image semantically. Scene parsing is the strict segmentation approach that attempts to partition the image into semantically meaningful parts and classify each image pixel into one of the pre-determined categories. Deep learning is one of the most advanced scene parsing [40] and can extract the underlying features directly from the raw image data, avoiding manual feature extraction. There are various technologies for deep learning applications, such as convolutional neural networks (CNN), fully convolutional neural networks (FCNN, [41]), pyramid scene parsing networks (PSPNet, [42]), and Deep Labelling for Semantic Network (DeepLabV3; [43]), etc. This paper uses DeepLabV3+ based on the Dilated FCN (Fully Convolution Network) framework for semantic segmentation. The proposed model, DeepLabv3+, is the fourth version of the Deep Lab series proposed by Google ([44]). It extends DeepLabv3 by employing an encoder-decoder structure to refine the segmentation results, especially along object boundaries. The overall architecture of the DeepLabV3+ model is shown in Fig 2. The collected SVP is inputted into the model and passes through the backbone network (which is the part marked as DCNN (deep convolutional neural networks) Atrous Conv(olution) in Fig 2). The encoder module encodes multi-scale contextual information by applying atrous convolution at multiple scales, while the simple yet effective decoder module refines the segmentation results along object boundaries. Finally, a pixel-wise classified street view image with semantic categories is produced. Meanwhile, it has the same size as the input image.

Generally, a deep learning model needs additional training to create a model suitable for scene segmentation and semantic understanding. Several datasets are available for training deep neural networks, such as ADE20k(https://groups.csail.mit.edu/vision/datasets/ADE20K/), PASCAL VOC (http://host.robots.ox.ac.uk/pascal/VOC/), etc. This paper uses the “cityscapes dataset” (https://www.cityscapes-dataset.com.) to train the model. The Cityscapes dataset is a large-scale database that focuses on semantic understanding of urban street scenes. The dataset consists of images labeled as objects for complex scenes in 50 different cities. The photos have the features such as a large number of dynamic objects, varying scene layouts, and varying backgrounds. Compared to other datasets, the cityscapes dataset contains the complexity of real-world landscapes [45], similar to the Baidu SVPs we use. The Cityscapes dataset defines semantic, instance-wise, and dense pixel annotations for 30 visual classes grouped into eight categories (flat, human, vehicle, construction, object, nature, sky, and void). It excludes 11 classes due to rare segments in streetscapes and leaves 19 classes for evaluation. In this paper, we use the default value (the number of finely annotated pixels) of 19 classes for semantic segmentation. The 19 classes and their associated categories are: the flat category including road and sidewalk, the construction category including building, wall, and fence, the object category including pole, traffic light, and traffic sign, the nature category including vegetation and terrain, the human category including person and rider, the vehicle category including car, truck, bus, train, motorcycle, and bicycle, and the final sky category.

**Fig 3:**
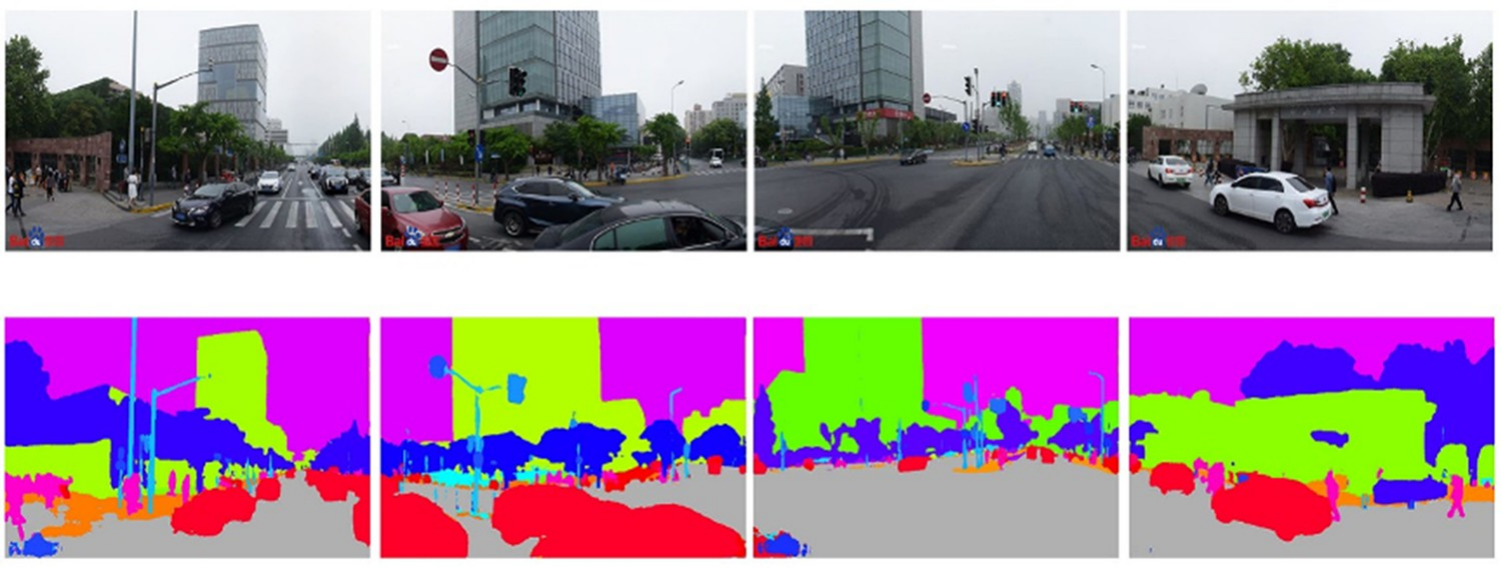
Semantic segmentation results of SVPs.

Based on the DeepLabv3+ algorithm and Cityscapes dataset, we complete the semantic segmentation for more than 7.8 million Baidu SVPs. That is, the segmentation (pixel-wise) of the images into multiple pixels sets of the sky, buildings, walls, and others (altogether 19 classes). Take the school gate of Tongji University in Shanghai as an example, and the semantic segmentation results are in Fig 3. The upper four SVPs are images with four different angles, and the lower four images are the pictures after semantic segmentation, respectively. The GPS point is the school gate of Siping Road, Tongji University, with the coordinates of (121.513046, 31.288205). Fig 3 shows that different objects in the pictures are clearly divided and given different colors. Among them, the color gray represents the road, yellow represents the sidewalk, blue represents vegetation, green represents buildings, red represents cars, purple represents the sky, purple represents pedestrians, blue-green represents poles, and so on.

**Fig 4:**
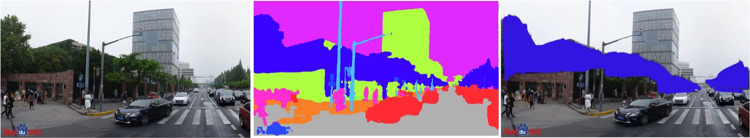
Greenery extraction results of two approaches.

**Fig 5:**
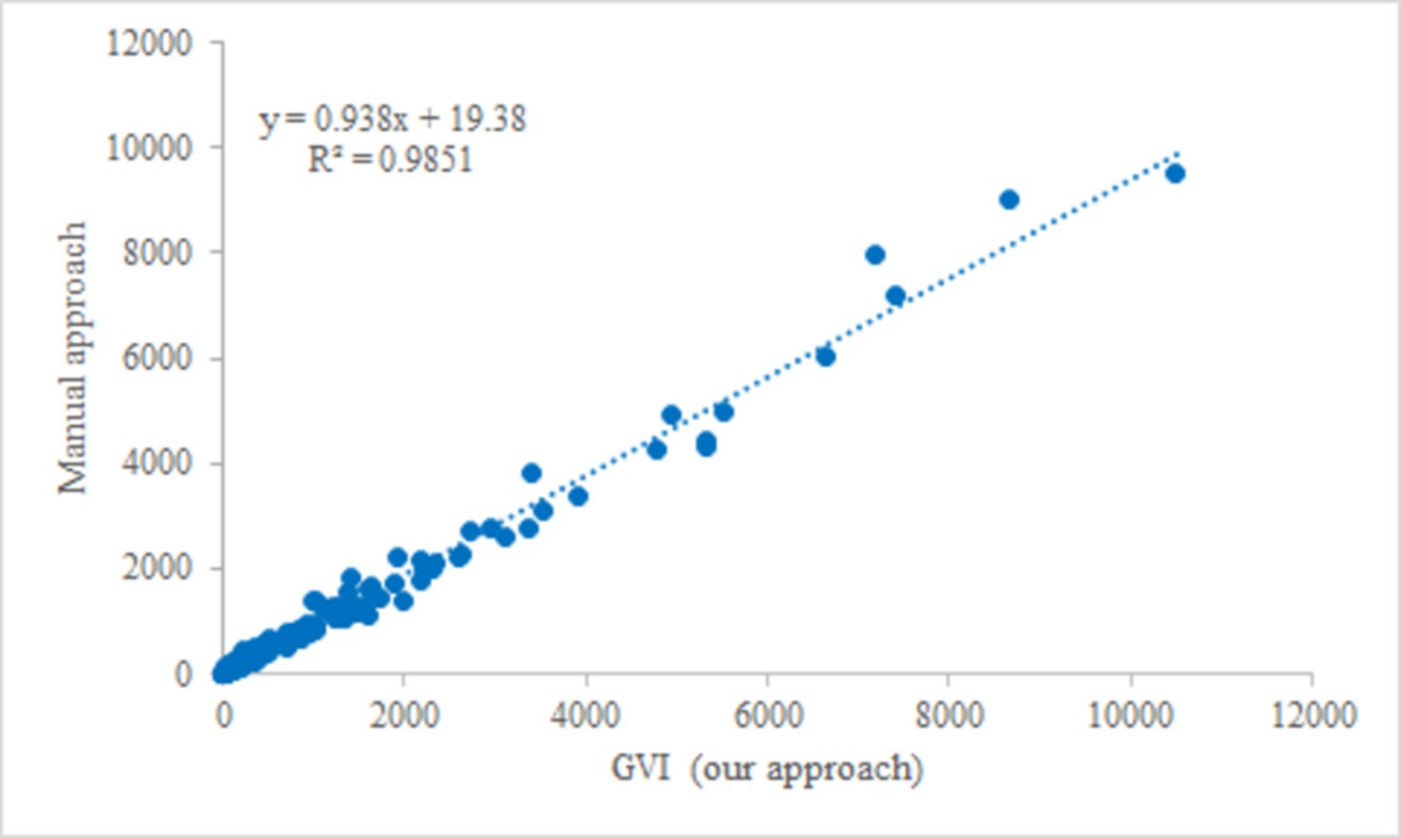
GVI results of two approaches.

Although literature review has shown that the DeepLabV3+ model is currently one of the most advanced techniques in recognizing SVPs in related studies, we carry out additional comparative assessments regarding the efficiency and accuracy of our approach. Fig 4 compares our approach and the manual approach using Adobe Photoshop. It is apparent that our approach detects more than the manual approach. In addition, referring to the method by Xia and others [40], we use linear regression to compare our results with those obtained manually. We randomly take SVPs and calculate the GVI (Green View Index) values for each image according to the measurement equation explained in the next section. Fig 5 shows the relationship between the GVI results of the two approaches. The correlation coefficient of the regression line is 0.938, with *R*^2^ = 0.9851.

### Measurement of neighborhood quality

As stated above, the neighborhood quality can be measured through four attributes: the level of traffic convenience, the urban architectural landscape, the greenery or ecological environment, and the scene visibility along the street. Referring to previous research, we use four indicators to evaluate neighborhood quality.

#### Measurement of the level of street facilities (*SC*)

Considering the available data, we use the street capacity (SC) to indicate the level of street facilities and the spatial accessibility of a city. Street capacity is usually measured by the physical scale of the road interface, such as the distance between buildings or structures (walls, fences) on both sides of the street [46], or the sum of secondary spatial scales such as motorized lanes, non-motorized lanes, and sidewalks [47]. Based on the image recognition information of various street-related objects, including motorized lanes, non-motorized lanes, sidewalks, pedestrians, vehicles, and so on, this paper first defines four street-related indicators. Then, we use the entropy method [48] to evaluate the street capacity based on the four indicators. The expression formula for the four street-related indicators is as follows:

$${PSW}_{i}=\sum_{k=1}^{{NUM}_{l}}{sidewalk}_{ik}$$

$${PRW}_{i}=\sum_{k=1}^{{NUM}_{l}}{road}_{ik}$$

$${CSW}_{i}=\sum_{k=1}^{{NUM}_{l}}\frac{{person}_{ik}+{rider}_{ik}+{bicycle}_{ik}}{{sidewalk}_{ik}}$$

$${CRW}_{i}=\sum_{k=1}^{{NUM}_{l}}\frac{{car}_{ik}+{truck}_{ik}+{bus}_{ik}+{motorcycle}_{ik}}{{road}_{ik}}$$

Where *PSW*_*i*_ is the proportion of sidewalks of city *i*, *PRW*_*i*_ is the proportion of roadways of city *i*, *CSW*_*i*_ is the capacity of sideways (non-motorized line) in city *i*, *CRW*_*i*_ is the capacity of roadways (motorized line) in city *i*. *NUM*_*l*_ is the total number of SVPs collected in city *i*, *road*_*ik*_ the pixel proportion of the motorized lane in the *k*th SVP of city *i*, *sidewalk*_*ik*_ is the pixel proportion of non-motorized lane and sidewalk in the *k*th SVP of city *i*. *person*_*ik*_, *rider*_*ik*_, *bicycle*_*ik*_, *car*_*ik*_, *truck*_*ik*_, *bus*_*ik*_, *motorbicycle*_*ik*_, is the pixel proportion of person, rider, bicycle, car, truck, bus and motorcycle in the in the *k*th SVP of city *i* respectively.

Here we apply Entropy Weight Method (EWM) to weigh the different impacts of these four indicators on the street capacity. It measures the degree of dispersion between variables, avoiding subjective evaluation and the information overlaps of variables. Generally, there are six steps when carrying out the EWM, including 1) data acquisition and collation, 2) data standardization to solve the homogenous problem existing in indicators’ values, 3) proportion calculation for indicators, 4) measurement of the information entropy [48] to solve the problem of quantitative measurement of information, 5) the calculation of the difference coefficient, which is negatively correlated with the entropy value, 6) after applying Python to realize entropy method, we get the weighted value for the four indicators as Table 1 shows. In this paper, we apply the weight of each indicator to calculate the street capacity of each city.

**Table 1:** The weight measurement of 4 indicators for evaluating the street capacity.

Level 1 indicator	Level 2 indicator weight	
Street capacity (*SC*_*i*_)	*PSW*_*i*_	0.2067
	*PRW*_*i*_	0.2526
	*CSW*_*i*_	0.2338
	*CRW*_*i*_	0.3069

#### Measurement of urban architectural landscape (*CD*)

We use the construction density to reflect urban development intensity, which relates to the city’s landscape directly. The construction density is the open space rate of the unit space and the density of the building coverage. Higher building density reflects the greater construction intensity of a city and thus the higher utilization degree of its urban space. The building density of an area is calculated by the percentage of building area to the whole area. Based on the information from the identification of SVPs, this paper calculates the building density with the following formula:

$${CD}_{i}=\sum_{k=1}^{{NUM}_{l}}{Building}_{ik}$$

*CD*_*i*_ is the construction density of city *i*, and *Building*_*ik*_ is the pixel proportion of buildings in the *k*th SVP of the city *i*.

#### Measurement of greenery environment (*GVI*)

Street greenery plays a vital role in enhancing the overall quality of a city [16]. The concept of “Green View Index (GVI)” was firstly proposed by Japanese scholars in 1985 [49] and was analyzed based on the ratio of green within the frame of vision. The green view index indicates the proportion of greenery in the street and reflects the degree of people’s visual perception of the surrounding greenery environment. It focuses on the three-dimensional composition and visual effect of urban greenery and thus can better reflect the quality of the public greenery environment from residents’ viewpoint [50]. Therefore, the Visible Green Index has a specific evaluation effect on environmental quality, urban planning, and residential value [51]. However, most early research mainly focuses on green coverage derived from satellite images or aerial photographs [16]. As Leslie and others [52] point out, the green indicators based on remotely sensed images are objectively measured and unable to represent neighborhood greenness perceived by pedestrians.

In 2009, Yang and others [53] use four pictures taken in four directions through an object-based image analysis approach and measure the VGI through the ratio of green area to the total area of the four pictures. It quantitively measures how much greenery a pedestrian can see from street level. After that, researchers have modified the GVI through various calculation methods (e.g., [40]). This paper refers to the method by Ki and Lee [32]. When considering the SVPs, VGI is the proportion of the number of pixels of greenery elements in SVP of the total amount of pixels of the picture. The calculation formula is as follows:

$${GVI}_{i}=\sum_{k=1}^{{NUM}_{l}}{Vegetation}_{ik}$$

*GVI*_*i*_ is the visible green index of city *i*, *Vegetation*_*ik*_ is the pixel proportion of vegetation in the *k*th SVP of city *i*. The value of *GVI*_*i*_ is high, when a lot of greenery is visible from a specific position.

#### Measurement of scene visibility (*DE*)

A street’s continuous scene visibility can satisfy people’s material, spiritual, psychological, behavioral, and other needs. It reflects a street’s original texture, making users feel the humanity care from the designers, thus promoting neighborhood quality [22, 23]. Public space of a city is mainly surrounded by various buildings. Scene visibility can be indicated by the spatial enclosure. The degree of enclosure is mainly composed of the proportion of street wall and of the sky [47]. In this paper, we include the sky proportion in reflecting the degree of spatial enclosure felt by pedestrians. We use the following formula to get the degree of enclosure in a city:

$${DE}_{i}={ALL}_{ik}-\sum_{k=1}^{l}{Sky}_{ik}$$

Where *DE*_*i*_ *is* the degree of enclosure of city *i*, *Sky*_*ik*_ represents the pixel proportion of sky elements in the *k*th SVP of city *i*. *ALL*_*ik*_ indicates all pixels in the *k*th SVP of city *i* (value is 1).

## Empirical analysis and results

### Basic regression

Because the Baidu SVPs are from the year 2016, we use income in the tourism industry (*TI*_*i*_) of the same year as a proxy measure to indicate the development level of the tourism industry in the city *i*. The corresponding regression empirical model is as follows:

$${TI}_{i}=\partial_{0}+\partial_{1}{QS}_{i}+\lambda X_{i}+\varepsilon_{i}$$ (1)

*TI*_*i*_ is the tourism income of city *i*; *QS*_*i*_ is the core explanatory variable of this paper and represents the neighborhood quality of city *i*, expressed by four indicators. Referring to the existing literature about the tourism industry (e.g., [54]), we adopt a series of control variables in city *i*, *X*_*i*_, including city’s level of economic development with indicator GDP (Gross Domestic Products), the level of service development with indicator the number of employees in the city’s service industry (POP3), the level of living standards with indicator the per capita income level (GZ), the degree of pollution with indicator the use of urban industrial dust emissions (GYFC).

Table 2 shows the regression results. Both estimated coefficients of two variables (*SC*_*i*_, *GVI*_*i*_) are significantly positive for the development of tourism development at level of 1% and 5% respectively. The results are consistent with those from Moniruzzaman and Páez [11] and Fok [18]. They insist that a high level of street convenience and the greenery or ecological environment tends to improve the city’s tourism industry. In addition, the estimated coefficient of the variable (*CD*_*i*_) has a significant inhibitory effect on tourism’s development. It is reasonable that people in modern society are tired of the high-density buildings and over-development of the city and prefer the natural environment. It also confirms the conclusion of Tyrväinen and others [14]. However, different from our expectation, there is no significant relationship between the variable (*DE*_*i*_) and the tourism income. That means, except for the variable scene visibility, all other three attributes of neighborhood quality significantly influence a city’s tourism industry. We can find in the following analysis part that the significance of four variable change when considering the city’s heterogeneity.

**Table 2:** Basic regression results.

	(1)	(2)
VARIABLES	TI	TI
SC	1.818***	1.888***
	(0.489)	(0.391)
GVI	12.42**	13.93**
	(6.136)	(5.606)
DE	-0.210	-1.350
	(0.897)	(0.818)
CD	-1.120**	-1.185**
	0.565)	(0.540)
GDP		5.66E-05
		0.000146
POP3		199.1*
		(104.1)
GZ		0.128
		(0.169)
GYFC		-0.0251
		(0.0284)
Constant	19,453***	9,232
	(1,597)	(8,697)
Observations	232	232
R-squared	0.661	0.702

Robust standard errors in parentheses

*** p<0.01

** p<0.05

* p<0.1

### Test of robustness

To test whether the above empirical results vary with the change of parameter settings, it is necessary to conduct a robustness test. We use two methods for robustness testing.

Firstly, we replace the explained variable tourism income (*TI*_*i*_) with the number of tourists (*TI*_2_*i*_) in the regression model. Columns (1) and (2) of Table 3 report the empirical results. The signs and significance of the explanatory and control variables are consistent with the baseline regression.

**Table 3:** Robustness test (replacement of the explained variables).

	(1)	(2)
VARIABLES	TI_2	TI_2
SC	1.806***	1.881***
	(0.531)	(0.433)
GVI	12.61*	14.23**
	(6.770)	(6.229)
DE	-0.0482	-1.276
	(0.975)	(0.904)
CD	-1.042*	-1.120*
	(0.622)	(0.593)
GDP		4.04e-05
		(0.000152)
POP3		223.8**
		(103.6)
GZ		0.187
		(0.190)
GYFC		-0.0136
		(0.0320)
Constant	23,161***	9,303
	(1,835)	(9,776)
Observations	232	232
R-squared	0.631	0.671

Robust standard errors in parentheses

*** p<0.01

** p<0.05

* p<0.1

Secondly, we delete some special city samples for robust regression. Cities in China present considerable differences due to their administrative levels.

Most of the cities in our sample are ordinary prefecture-level cities. However, there are about 39 special cities with various administrative levels such as the provincial capital city, municipalities directly under the central government, sub-provincial cities, special economic zone cities, and cities listed in the national social and economic development plan. Because the administrative level indicates heterogeneity in resources capacity and policy-making rights which might influence the development of urban tourism, the difference at the city level may bias the regression results. Column (1) in Table 4 shows the regression results after deleting the special city samples. Column (2) in Table 4 shows the results after changing the explained variable with the number of tourists. Both results show that the signs and significance of the explanatory and control variables are consistent with the baseline regression.

**Table 4:** Robustness test (delete special city samples).

	(1)	(2)
VARIABLES	TI	TI_2
SC	5.255**	4.927***
	(2.043)	(1.846)
GVI	5.984**	5.488**
	(2.477)	(2.285)
DE	-0.684	-0.329
	(1.452)	(1.263)
CD	-55.78***	-50.92***
	(19.61)	(17.95)
Constant	20,683***	16,800***
	(2,441)	(2,079)
Control Variable	Yes	Yes
Observations	193	193
R-squared	0.474	0.487

Robust standard errors in parentheses

*** p<0.01

** p<0.05

* p<0.1

The above two tests show that the signs and significance of the coefficients of the main variables are consistent with that of the basic regression, indicating that our empirical results are robust.

### Threshold test

Existing literature has demonstrated that China is a vast country with distinct heterogeneity in various aspects among cities. That is to say, heterogeneities in different cities might lead to differences in the intensity of the influence from neighborhood quality. To make a reasonable explanation for the heterogeneity, this paper further explores the threshold effect with consideration of the economic level measured by the GDP. Table 5 shows that the economic level in the 39 special cities is significantly higher than that of the ordinary cities.

**Table 5:** Descriptive statistics of economic levels in different types of cities.

	object	Mean	Std.	Min	Max	Obs
**Economic level**	**Special city**	0.76	0.565	0.121	1.961	39
	**Ordinary city**	0.219	0.281	0.015	1.552	193

Table 6 shows the test for the threshold effect with the economic level as the threshold variable and the neighborhood quality as the core independent variable. The P-value of 0.043 at 5% significance level indicates a significant single threshold effect with a threshold value of 0.248. In addition, the result that the P-value of 0.279 is not significant reflects that the model has no double threshold effect. It means that only after the city’s economic level reaches the threshold of 0.248, the neighborhood quality begins to exert its impact on the tourism industry.

**Table 6:** Threshold effect.

The threshold variable	The threshold model	*P*	*BS*	*threshold*	*LOWER*	*UPPER*
Economic level	Single threshold	0.043**	5000	0.248	0.247	0.248
	Double threshold	0.279	5000	0.248	0.247	0.248

Furthermore, Table 7 shows the result of the threshold regression. If the economic level is less than the threshold (0.248) in Column (1), all four attributes of neighborhood quality have no significant effect on the tourism industry. Conversely, if the economic level crosses the threshold value (0.248) in Column (2), the results are basically same as that of the basic regression in Table 2. The results here testify the existence of cities’ heterogeneity.

**Table 7:** Threshold test.

	(1)	(2)
	GDP<0.248	GDP> = 0.248
VARIABLES	TI	TI
SC	2.605	1.870***
	(1.651)	(0.477)
GVI	16.74	14.25**
	(19.46)	(6.630)
DE	-0.625	-1.193
	(2.077)	(0.970)
CD	-0.546	-1.096*
	(2.395)	(0.651)
GDP	0.00123**	7.22e-05
	(0.000565)	(0.000200)
POP3	178.4	210.4*
	(180.1)	(121.2)
GZ	0.439***	0.117
	(0.152)	(0.428)
GYFC	0.0935**	-0.0348
	(0.0446)	(0.0338)
Constant	-8,979	11,337
	(9,642)	(21,594)
Observations	141	91
R-squared	0.171	0.697

Robust standard errors in parentheses

*** p<0.01

** p<0.05

* p<0.1

## Discussion

Previous studies have explored that the improvement of neighborhood quality has an influence on urban tourism. However, most assess the neighborhood quality subjectively and on a small scale. The lack of quantifying neighborhood quality leads to doubts about the applicability and reliability of the research conclusions. Traditional methods such as case studies, questionnaires, and field surveys are time-consuming and not applicable to large-scale studies. Although previous literature has applied satellite remote sensing images to quantify environment characteristics (e.g. [55]), these images are from a bird’s eye or overhead view, which is different from our study that requires a visitor’s or pedestrian perspective. Therefore, the application and methodology of Baidu SVPs in this paper meet the accessibility of large-scale detailed data and the possibility of measuring the neighborhood environment in a quantitative way, ultimately making our discussion of the impact of the neighborhood quality on tourism more accurate and reliable. To the best of our knowledge, this paper is the first to combine SVPs, deep learning techniques, and regression models to examine the influence of neighborhood quality on tourism development in almost all Chinese prefecture-level cities.

This paper contributes to the literature on urban planning. Most previous researches employ questionnaire or field research to explore the influence of neighborhood quality. Our findings in this paper provide new insight into how four attributes of neighborhood quality affect the tourism industry, thereby enriching the existing discussion about urban design and planning. In addition, our study provides a methodological reference for employing SVPs and deep learning techniques in economic study, especially the use of large amounts of data at the micro-level.

Our results are of great significance for policymakers in improving China’s urban construction and urban tourism economy. In recent years, the regional integration process in China has accelerated significantly. As the basic unit of the city, the city block carries the city’s main public space. The neighborhood quality of a city is closely related to the city’s attractiveness and thus its economic development issue. Meanwhile, “ecological civilization” has become one of the national strategies and also the primary guidance for spatial development in China. Under these circumstances, improving neighborhood quality in a city has become an essential strategy for local governments to participate in the new urban development process. More detailly, this article presents some policy implications for effectively promoting local quality in the aspects such as street capacity and greenery environment. In addition, it reminds people to avoid dense construction in a city. However, the existence of such impact requires a threshold point, namely, it depends on a city’s economic level. If the overall economic level of a city reaches a certain degree, its urban spatial design should gradually shift from “scale expansion” to “stock optimization”. For cities with relatively low economic levels, the practice of promoting the urban economy through infrastructure investment and construction is reasonable. Meanwhile, they need to have enough space for future urban space design, such as greenery and a reasonable layout of urban buildings and streets.

Although the SVP is a valuable data source for assessing the urban environment, some scholars have raised its limitation in recent years. Firstly, almost all existing studies on Chinese street maps do not involve multi-temporal analysis and are carried out for a specific time, e.g., the year 2016 in our study. However, a time series of data can better reflect the dynamic change of neighborhood quality and thus the influence across time. The main reason lies in our acquired Baidu SVPs, captured in a single day or a short period and lacking continuous temporal coverage ([56]). Although Baidu has added the “time machine” function in its online query system that provides multi-temporal SVPs, users are not able to acquire these historical SVPs through the API access. The study by Yu and others ([57]) is one of the few studies focusing on temporal changes. The research uses Baidu SVP to assess the dynamic changes in urban greening in 2014 and 2019. However, their study is limited to one Chinese prefecture-level city. In addition, the SVP is criticized for its bias in spatial coverage and street accessibility. Chen and others ([58]) argue that the vehicle used to take the SVPs cannot reach some streets or alleys in the city. Especially for tourists, the places that attract them may not be reflected on the street view map. Sometimes, the weather conditions or weather seasonality at the time of photo taking, such as lighting and solar radiation ([56]), also limit the quality of the photo and thus the precision of the recognition. Under this circumstance, some other data sources need to be added to ensure the accuracy of the analysis. Furthermore, the subjective perception measures based on the real environment and based on the street view may not be consistent. The study from Feng and others ([59]) points out that the Baidu SVPs and the real site are relatively consistent in the subjective perception assessment of street quality while significant differences exist in the ambiance perception. Yue and others ([60]) also acknowledge the importance of human visual perception of the urban environment, in addition to the physical environment. Future research would be made to combine other techniques or information requisition sources such as questionnaires for accurate assessment.

In addition, existing literature has applied various indicators to explain the concept of neighborhood quality. This paper describes the concept from four attributes. However, there exist other variables relating to the quality of the neighborhood, such as demographic makeup [61], psychological perception [4] including safety [16], wealth and boring, etc, urban commerce distribution [39], social inequalities [62] and perceived scene complexity [63]. Considering the tourist-related characteristics and the data availability from VSPs, this paper limits to the four indicators. Future related studies can focus on the above-mentioned aspects and yield more significant implications. For example, our future study tries to use POI (point of interests) data obtained through Baidu, hoping to improve the assessment of neighborhood quality. Also, the definition and measurement of some indicators for neighborhood quality in our paper seems simple to some degree. Although our evaluation of the first indicator, the level of street facilities, is relatively comprehensive, the measurements of the other three indicators only rely on the proportion of pixels. One of the reasons is that our methodological analysis is relatively new, and not much literature can be referenced, except for the greening rate. The other reason is the limitation of the VSP identification. Therefore, the limited measurement might weaken the effectiveness and robustness of such an analysis. Future research will benefit from the advances in VSP-identification technologies.

## Conclusion

This paper fills the research gap by using Baidu SVPs and deep learning to evaluate quantitatively four attributes of neighborhood quality, namely street facilities, architectural landscape, green or ecological environment, and scene visibility. Based on the more than 7.8 million Baidu SVPs of 232 prefecture-level cities in China, the paper attempts to investigate the influence of neighborhood quality on the development of the tourism industry in a city. Empirical study finds that: (1) The improvement of the neighborhood quality, in general, promotes the development of the tourism industry in a city. More specifically, the level of street facilities with an indicator of street capacity and greenery environment with indicator GVI significantly positively influence tourism. Conversely, higher construction density (*CD*) has significant inhibitory effects on the development of the tourism industry. (2) These effects have the threshold effect when we take cities’ heterogeneity into account, such as the urban economic level of a city. Only when the economic level crosses the threshold value of 0.248, neighborhood quality has significant impact on the urban tourism industry.

Compared to previous related studies assessing neighborhood quality subjectively and on a small scale, our study shows that the Baidu SVPs and deep learning techniques can be regarded as a reliable tool for evaluating neighborhood quality for a large-scale study area. The methods proposed in this study could be applied to related research areas in other countries and regions, such as land use and built environment, health and wellbeing, urban modeling and demographic surveillance, etc. ([64]).

## Supporting information

S1 File (DTA)

S1 Dataset (DOCX)
