# Supplementary material for: The influence of neighborhood quality on tourism in China: Using Baidu Street View pictures and deep learning techniques
Source: PLoS One. 2022 Nov 3;17(11):e0276628. doi: 10.1371/journal.pone.0276628 (PMC9632836; doi:10.1371/journal.pone.0276628)
Supplement: S1 Dataset — (DOCX) [file pone.0276628.s002.docx]

This dataset supports the paper “The influence of neighborhood quality on tourism in China: using Baidu Street View Pictures and deep learning techniques” (not published yet, we will add the link after published).

This is a cross-sectional data for 232 prefecture-level cities in China in year 2016. It includes not only the variables used in the paper, but also other city-level variables.

The following variables: “pole, traffic_light, vegetation, traffic_sign, terrain, sky, rider, car, person, truck, bus, train, motorcycle, bicycle” describing the characteristics of a city and are acquired and recognized from the Baidu Street View Pictures. The detailed procedure needs to refer to the paper.

More than 7.8 million Baidu Street View pictures (SVPs) in China are collected to measure the neighborhood quality on city-level. The SVPs came from the Baidu Map open platform (<http://lbsyun.baidu.com/>). Baidu Street View and Tencent Street View have covered most cities in China and are now the primary street view data sources in mainland China. Map open platform provides free online access and an API (Application Programming Interface) for downloading Baidu SVPS. (Baidu SVPs API: http: / / api.map.baidu.com/panorama/v2?ak=*&width=512 &height=256 &location=116.313393,40.04778&fov=180).

After getting the SVPs, we need to have a precise semantic segmentation for the different physical features in each image and assign a category label to each pixel in the image.

We usesDeepLabV3+ based on the Dilated FCN (Fully Convolution Network) framework for semantic segmentation. The proposed model, DeepLabv3+, is the fourth version of the Deep Lab series proposed by Google.

We then measure the neighborhood quality through the data recognized from SVPs.

Other data to evaluate the city-level variables are from the China City Statistical Yearbook coedited by the National Bureau of Statistics of China (NBS).
